# Redrawing the frontiers in the age of post-publication review

**DOI:** 10.3389/fgene.2015.00198

**Published:** 2015-06-05

**Authors:** David W. Galbraith

**Affiliations:** ^1^BIO5 Institute, University of Arizona, Tucson, AZ, USA; ^2^School of Plant Sciences, University of Arizona, Tucson, AZ, USA

**Keywords:** post-publication review, scientific misconduct, internet publication, scientific reproducibility, fraudulent data

## Abstract

Publication forms the core structure supporting the development and transmission of scientific knowledge. For this reason, it is essential that the highest standards of quality control be maintained, in particular to ensure that the information being transmitted allows reproducible replication of the described experiments, and that the interpretation of the results is sound. Quality control has traditionally involved editorial decisions based on anonymous pre-publication peer review. Post-publication review of individual articles took the lesser role since it did not feed directly back to the original literature. Rapid advances in computer and communications technologies over the last thirty years have revolutionized scientific publication, and the role and scope of post-publication review has greatly expanded. This perspective examines the ways in which pre- and post-publication peer review influence the scientific literature, and in particular how they might best be redrawn to deal with the twin problems of scientific non-reproducibility and fraud increasingly encountered at the frontiers of science.

## Introduction

**FIGURE 1:**
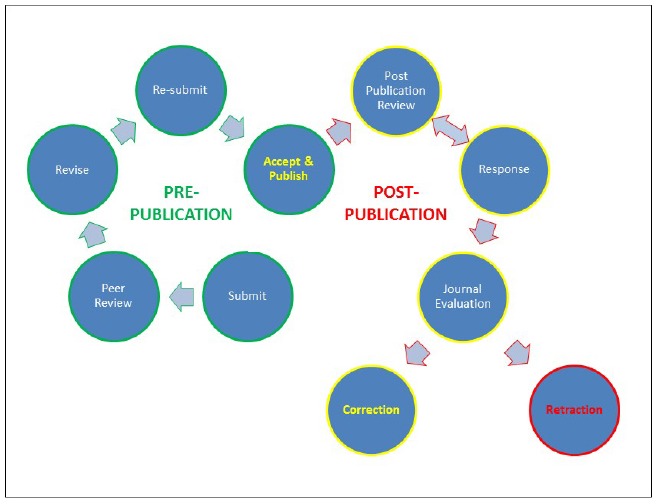
**Schematic illustration of the pathway of conventional pre-publication review and of emerging post-publication review.** The extent and impact of post-publication review has been greatly enhanced by the communications revolution associated with the development of computer technologies and the internet.

The procedures for publication of written works have been elaborated from the time of William Caxton, and, using English, are the world-wide standard for scientific communication. Anonymous pre-publication peer review is recognized as essential for maintenance of the high standards necessary to advance scientific understanding. Over the years, various models for funding the distribution of published information have emerged, and recent advances in computer technologies have greatly reduced costs, and increased the numbers of outlets available for scientific publication. Keeping abreast of emerging concepts and discoveries is becoming increasingly problematic. Since publication is central to advancement in an academic career, and given that specific journals occupy hierarchies of preeminence, largely based on citation index, coupled to a general increase in competition for increasingly restricted funding, unprecedented pressure has been placed on traditional pre-publication quality control (Figure 1). This pressure is compounded by the emerging recognition of issues of scientific reproducibility, as well as issues associated with scientific fraud. Post-publication review now provides a powerful means to identify and correct errors of quality control, of experimental design, and of data interpretation. It also allows facile detection of malfeasance, and provides a structural framework encouraging ethical behavior during publication, including deterrence through the real prospect of punishment of malefactors.

## Post-Publication Review

Post-publication review is a recent development emerging as a consequence of the technological revolution of the internet. As a concept, post-publication review has been around from the very beginning of scientific publication. Discussion and debate is, and always will be, a central part of the scientific method, its strength being that this allows establishment of consensus, as well as identification of conflicting results and competing ideas. From these, further experiments can be proposed and performed, leading to a more sophisticated understanding of the natural world. Post-publication review also serves to identify and remove flawed data, to correct misattribution, and to provide useful links to information emerging post-publication. Some journals provide opportunities for post-publication review, generally under non-anonymous conditions (PLoS ONE, Science, and Nature, for example).

Given the fact that internet-based communication is essentially free, and that most if not all of the scientific literature is accessible in electronic formats, it is logical that websites have recently emerged that provide anonymous forums for post-publication review. PubPeer^1^ is a particularly noteworthy example.

Describing itself as The Online Journal Club, PubPeer provides a website at which scientists can upload anonymous comments concerning published articles, notification of these comments being automatically transmitted to the authors, along with the opportunity to respond. PubPeer states that “The chief goal of this project is to provide the means for scientists to work together to improve research quality, as well as to create improved transparency that will enable the community to identify and bring attention to important scientific advancements.” This site indeed hosts discussions of genuine differences of opinion concerning experimental design or interpretation, of science funding and other policy issues, and of the equitable functioning of pre-publication peer-review. However, a large proportion of the postings center on identification of potential scientific malfeasance within publications.

The power of PubPeer is particularly enhanced by crowd-sourcing, since it takes little effort for multiple anonymous individuals to identify and post specific problematic issues. The remarkable speed with which the entire published output of an individual scientist can be analyzed emphasizes the power of crowd-sourcing. What also is remarkable is the extent of apparently fraudulent behavior across the field of science as a whole, and across a very wide range of journals, from the most prestigious to the most obscure. Inappropriate image duplication, which seems to comprise the largest subset of current postings, is particularly seen for gel analyses, but also extends to microscope images, and even flow cytometric histograms. Given the remarkable ability of the human eye to rapidly detect identical patterns, it is surprising that any scientist would expect data manipulations of this type to remain undetected for long. Now, since all publications end up eventually in freely-accessible electronic format, the new reality is that data manipulation will *never* remain undetected. Machine learning is also not far behind humans in terms of face recognition (Taigman et al., 2014), and automated analysis of images from networked groups of scientists has already led to detection of widespread malfeasance (Abbott, 2013). Biological datasets other than images can be subjected to statistical analysis to detect illegitimate manipulation, since these datasets necessarily contain noise derived from the means of measurement and from the properties of the system under study (Yong et al., 2013), and this noise should not display unusual characteristics.

Another popular website, Retraction Watch, currently funded by the Macarthur Foundation, deals with post-publication review in a different manner, instead presenting, as journalism, the end results, largely negative, of this process. Retraction Watch incorporates information from sources beyond the primary scientific literature, as well as providing editorials on emerging topics. Of recent concern has been the underlying causes of retraction, the willingness of journals to enforce retraction, whether or not retraction rates are changing, and the overall cost to society. Comments on individual stories are allowed from external readers; these can be anonymous and, in some cases, extensive. NCBI now also provides the ability to provide non-anonymous comments to archived journal articles through PubMed Commons.

As for any new discussion forum, individuals are still learning post-publication review etiquette. Guidelines provided by PubPeer and Retraction Watch (as well as PLoS ONE, Science, and Nature) aim to restrict obviously inappropriate postings using moderators. Since post-publication review is now widespread across many journalistic outlets beyond science, commenters are generally aware of the forms of unacceptable behavior, including general “trolling” (obnoxious postings designed to upset), “sock puppetry” (presentation of one side of an argument via impersonation of multiple anonymous individuals), identity theft, and use of the “Gish Gallop” (rapid-fire presentation of multiple spurious arguments to overwhelm debate). Finally comments that might be interpreted as libelous are removed. One recent PubPeer thread has discussed the desirability of establishing an editorial board. At present, the arguments in favor of such a board appear to be outweighed by free-speech concerns as well as the value of anonymity. Others are in favor of transparency, arguing that this allows evaluation of the credentials of the commenters, yet at the same time expressing concern regarding the effect of social dominance and stereotypical discrimination (Bastian, 2014). Importantly, although the opportunity for individuals to identify themselves is available, the ability to remain anonymous on PubPeer and Retraction Watch seems desirable to protect commenters from retaliation, particularly early career scientists.

A final path to post-publication review is that taken by the individual whistleblower (see, for example Yong et al., 2013), but it seems likely that this approach will be subsumed by PubPeer and Retraction Watch given the greater efficacy of crowdsourcing.

## How Bad is the Situation?

A central tenet of scientific investigation is that the results should be reproducible. Work that is not reproducible should be expunged from the scientific literature, since it serves no value at best, and at worst can adversely influence the pursuit of knowledge. Furthermore, studies found to be non-reproducible may be cited by secondary publications at higher rates than those found to be reproducible (Begley and Ellis, 2012). Prinz et al. (2011) and Begley and Ellis (2012) have provided widely-discussed commentaries concerning the low rate of reproducibility of landmark experiments in preclinical cancer research. This lack of reproducibility may explain in part the low recent rate of development of effective novel drugs and therapies. Post-publication review clearly has a critical role to play in verifying reproducibility, since beyond fraud, it can identify improper experimental design, inadequate descriptions of experimental manipulations, and unrecognized sources of variation (Galbraith, 2006). Post-publication review can also address other areas of concern, including inadequate statistical design (Ioannidis, 2005), and the problems associated with use of *P*-values, rather than effect sizes and confidence intervals, to describe statistical significance (Nuzzo, 2014).

One way to quantitatively analyze the major factors contributing to irreproducibility is through retrospective tracking of the reasons stated for retractions (Fang et al., 2012; Casadevall et al., 2014). Out of a total of about 25 million articles indexed in PubMed over the study period, only 2,047 were identified as having been retracted (Fang et al., 2012). A small minority (21.3%) of retractions were due to error, with the majority (67.4%) being attributed to scientific misconduct. The proportion of scientific articles retracted because of fraud appears to have increased by almost 10-fold since 1975. On the other hand it seems that a relatively small number of individuals are responsible for a major proportion of these retractions. Thus, 38 research groups having a total retraction rate of five or more articles during the sample period accounted for almost one-half of the total retractions for fraud or suspected fraud; for groups having 10 or more retractions, most were due to fraud. Fraud can be overt or can be more subtle, for example in selective omission or inclusion of datasets in the final publication. Statistical methods themselves can be gamed, in the form of *P*-value manipulation (“P-Hacking”), which involves selecting datasets and/or methods of statistical analysis until non-significant results attain significance (Head et al., 2015). Meta-analysis can detect P-Hacking, as revealed by unexpected deviations of *P*-value distributions around a value of 0.05 (Head et al., 2015). This activity, as compared to the overt fraud described previously, seems quite widespread, perhaps since it is not clearly recognized by the scientific community as a form of fraud. Problems associated with fraud are also magnified by the general approach taken in experimental laboratory science, in which the investigator starts with a proposition that they generally feel is correct, and then designs experiments to verify the predictions of this proposition. The danger comes when data is massaged, or simply made up, to provide these verifications, since it seems inevitable that all models for the living world will at some point in time turn out to be incomplete (Galbraith, 2006). Further, the step is a short one from duplication of “control loading” lanes, an easy temptation when experiments are repetitious and the technology is unremarkable and generally reproducible, to full-scale manufacture of fraudulent data. The degree to which photoshopping of gel data can be readily recognized, and still persists in the most recently published journal articles, is astonishing (see text footnote 1).

**FIGURE 2:**
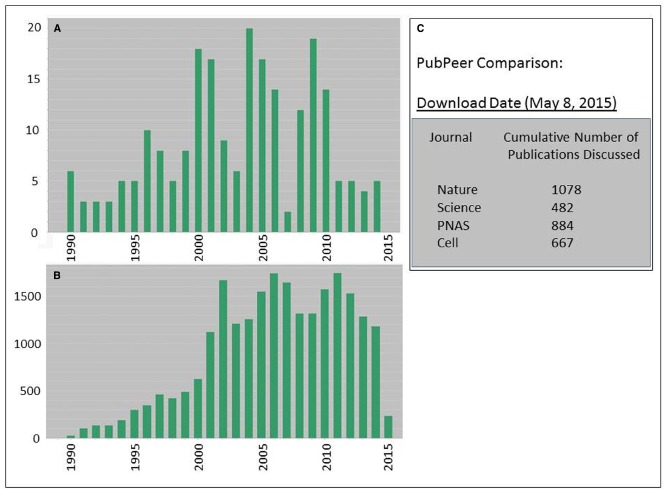
**Tracking the impact of questionable research through retraction notices (A,B) and post-peer review (C). (A)** A data search of Thompson-Reuters Web-of-Science (WOS) using the following Search Criteria: PUBLICATION NAME: (Science or Nature or Cell or Proceedings of the National Academy of Sciences of the United States of America) AND YEAR PUBLISHED: (1990–2015) Refined by: DATABASES: (WOS) AND DOCUMENT TYPES: (ARTICLE OR LETTER OR REVIEW OR EDITORIAL) AND DOCUMENT TYPES: (RETRACTION). Total items (unretracted plus retracted): 635,032. Total retracted items: 223. **(B)** Citations to the 223 retracted items in WOS appearing over the analyzed period. The sum of the times cited was 23,662, from 21,939 citing articles, resulting in a h-index of 90. **(C)** Comparative activity in PubPeer for the same four journals.

Differences do appear to exist between the seemingly low overall rates of retraction reported in the published literature (Fang et al., 2012) and a much higher level of post-publication review activity in PubPeer. The first panels of Figure 2 illustrate a search using Thompson-Reuters Web-of-Science scanning four major publications (Nature, Science, Cell, and PNAS) for total publications and retractions (Figures 2A,B) over the period from 1990 to 2015. Figure 2C cites the current cumulative activity for these four journals in terms of total numbers for papers that have been subjected to post-peer review at PubPeer. Although these datasets are not easy to compare directly, they imply a degree of disconnection between the effectiveness of pre-publication review, the identification of post-publication concerns, and the ultimate fate of questionable articles. The fact that retracted articles from the four top-ranked journals in this specific sample were cited widely and at high levels (even generating a h-index score of 90!; Figure 2B) highlights the way that false information can quickly metastasize through the body of scientific knowledge. Others have noted a correlation between journal impact factors and retraction rates (Liu, 2006; Cokol et al., 2007; Fang et al., 2011), as well as a trend to higher levels of retraction over time (Cokol et al., 2008).

Of course, the fact that an article has been retracted does not necessarily imply misconduct, or that the work is not reproducible; it may simply indicate the conclusions that were drawn were incorrect. For example, it is now known that many specific animal cell lines have been contaminated and overgrown by HeLa cells (reviewed by Neimark, 2015). Retractions of course should also be made if subsequent experiments invalidate the conclusions of the publication. Two recent high-profile examples include the observation of poor correlations between morpholino-induced and null mutant (CRISPR/Cas9) phenotypes in zebrafish (Kok et al., 2015), and the observation that a CRISPR/Cas9 null mutation in the ABP1 gene of *Arabidopsis* displays no developmental or auxin-related defects (Gao et al., 2015). In both cases, off-target effects are the likely explanation, and this may well invalidate the general use of morpholino nucleic acids (and to a certain extent, insertional mutagenesis, and RNAi approaches) for negative modulation of gene expression. In the latter case, the incorporation of ABP1 into elaborate pathways of auxin signal transduction, resulting in additional high-profile publications, is hard to reconcile with the phenotype of the ABP1 null, and the fall-out within the field of auxin signaling in general may well be substantial.

## The Costs of Scientific Fraud

Papers retracted due to misconduct from 1992 to 2012 accounted for approximately $58 million in NIH funding, or less than 1% of the NIH budget (Stern et al., 2014). Although this proportion is small, it represents only the direct costs of grants associated with specific fraud, and does not take into account the cost of any subsequent fruitless work based on the original false information, particularly that within private industry. False positive results in the literature can be very persistent, since there is little incentive or funding to replicate published results, and even when replication is done, early positive studies often attract more attention than later negative ones (Head et al., 2015).

A pernicious effect of data manipulation directly affects the integrity of the community of scientists. When data manipulation allows authors to accelerate publication of work that lacks control, but that subsequently turns out to be correct, the impact of the fraud can be downplayed. In essence, the fraudster has calculated the probability of the work being reproducible, based on limited primary observations but also on previous knowledge, to gain publication priority. The damage in this situation is not limited to that of persistent false positives, but extends to competing principal investigators that are scooped (particularly early career scientists), and to unwitting co-authors.

The political cost of scientific fraud could be disastrous. Particularly in the United States, science has become a political football, having lost traditional bipartisan support as an objective arbiter of the state of the known world. Anything that diminishes the respect that is due to the scientific method would be extremely dangerous.

In terms of retraction, the path to publishing contradictory results is not necessarily an easy one (Vaux, 1998, 2013). The editors of high profile journals increasingly have to negotiate conflicts-of-interest arising from the financial reward associated with maintaining journal profiles, the attraction of being first to publish groundbreaking work, the negative consequences of retractions, and, perhaps inevitably, that they will have to deal with legal liability emerging from inadequate peer-review. Since a large majority of scientific publications arise from work funded by the public sector, the move toward accountability, with accompanying reformation of editorial activities, if needed, appears inexorable.

## Moving Forward

The first, and most important recommendation is a general one. Scientists must recognize and accept the impact of changes to our world driven by our unprecedented ability to derive, manipulate, store, and distribute data. Data privacy is an obsolete concept, and we must adapt to a world through which we move, shedding data constantly for all to observe. Post-peer review is here to stay, and although it may from time to time appear anarchic (Faulkes, 2014), its potential for both distributing scientific information and improving its quality are considerable, and the potential will continue to grow.

Another recommendation is more practical: relative to publication, all primary data should be provided as supplemental files in unmodified form. Guidelines for publication should universally include prohibitions on selective excision and reconstitution of parts of images. Improved handling of statistical issues and experimental design should be implemented. Movement in this direction is exemplified by the Journal of Cell Biology Dataviewer, which provides a location for uploading the original data that supports published papers.

Further along this line is the possible use of federal standards to curate data streams in published papers. In the US, this could well involve *Title 21 CFR Part 11* from the Code of Federal Regulations that establishes United States Food and Drug Administration (FDA) rules on Electronic Records and Electronic Signatures. Part 11 defines the criteria under which electronic records and electronic signatures are considered to be trustworthy, reliable, and equivalent to paper records [Title 21 CFR Part 11 Section 11.1 (a); Wikipedia].

The rules in 21 CFR Part 11 were designed with the biotechnology, pharmaceutical, medical device, and biotechnology industries in mind, since these are directly regulated in the United States by the FDA. Electronic records that are 21 CFR Part 11 compliant have an electronic timestamp that defines the signer name, as well as the date and time and the meaning of the signature. The FDA recommends an audit trail for the associated electronic records, to provide certainty that no one else has accessed these records without permission. If Title 21 CFR Part 11 were to be used, data acquisition and manipulation software would have to become compliant with this pipeline. Ironically, this would ensure that electronic records used to generate paper records are reliable, the exact converse of the original reason for establishing Title 21 CFR Part 11!

Critical reexamination also is required at the level of pre-publication peer-review, to ensure this process is as transparent as possible, to ensure accountability at the level of the journal editorial boards, and to eliminate persistent suspicions of favoritism during the review process. Publishing the reports of the referees, and the responses of the authors, in the supplemental materials could improve the review process. It would show the extent to which reviewers provided intellectual input, and would also help eliminate sundry additional malfeasances associated with publication, such as h-index manipulation (Bartneck and Kokkelmans, 2011). Additional transparency at this level could provide identities of reviewers but, again, this might raise issues of retribution. A further emerging option is that of preprint publication, in which draft articles and the associated data are provided prior to formal submission for publication (see, for example the biorxiv preprint server for biology). This approach, widely employed in physics, allows immediate community input prior to the formal peer-review process. After about 18 months of operation, biorxiv appears to be gaining acceptance^2^, and it seems that the worry of losing priority for publication in top journals following publication of a preprint is not warranted. Of course, this approach raises questions of “what constitutes publication” which ultimately will be settled in courts of law.

Finally, from the preceding discussion, although it is clear that the process of scientific publication is undergoing change that is both revolutionary and evolutionary, and it is unclear as to what might evolve as the preferred form of publishing, we must remember that we work within the public trust. There is increasing political pressure on science, from all directions, to accommodate the unfortunate consequences of anthropogenic activities. We cannot risk, as scientists, being accused of malfeasance in discovery. Thus, post-publication review is something in which all true scientists should fully participate. Furthermore, we should not excuse senior scientists that are found to be complicit in fraud. Given a near exponential growth of scientific productivity across the world, as reflected in the growth of published literature, one can reasonably conclude that everything that can be discovered, will be discovered, likely sooner than anticipated, and without the contributions of any individual. Thus, any scientist is ultimately disposable, whatever their level and qualifications. Elimination of fraud serves the common good both in terms of providing increased efficiency of resource allocation, important now, and providing role models for entering scientists, critical for the future.

### Conflict of Interest Statement

The author declares that the research was conducted in the absence of any commercial or financial relationships that could be construed as a potential conflict of interest.

## Footnotes

1 www.pubpeer.com

2 http://scim.ag/bioRxiv1yr
